# A Radium Week by Artizan Workers

**Published:** 1914-06-13

**Authors:** 


### NORTHAMPTON HOSPITAL AND A NATIONAL EFFORT.

### A Radium Week by Artizan Workers.

The radium difficulty has called forth the whole-hearted co-operation of the working classes throughout Northamptonshire on behalf of the Northampton General Hospital. In connection with this institution there is a Hospital Week Committee with organised centres throughout the county, which collects a large and increasing sum every year in support of the Northampton Hospital. This movement has the good fortune to be under the organising direction of a very able secretary, Mr. Charles H. Battle, who is full of useful and attractive ideas. Mr. Battle once stood by the bedside of a woman in middle life who was afflicted with cancer which was declared incurable. The case was full of pathos from the fact that the patient had two young daughters who their mother was fearful by her death might be left alone in their early teens, so she prayed that she might be spared long enough to remove this danger from them. This case made a deep impression upon Mr. Battle, and he mentally resolved, should a cure ever be discovered, that no effort on his part should be spared to place it within the reach of every suitable case.

When radium came to be regarded as being capable of affording cancer patients relief, Mr. Battle evolved a scheme to provide Dr. W. M. Robson, the physician in charge of the electrical and *x*-ray department of the Northampton General Hospital, with an adequate supply of radium. Mr. Battle hit upon the plan of a Radium Week, to extend from May 10 to May 16, 1914, inclusive, during which the whole-hearted, practical support of every resident throughout the town and county of Northampton should be invited on behalf of a supreme effort to raise at least £1,000 for the purchase of a supply of radium without delay. The Hospital Week Committee, with the support of the Mayor and Mayoress of Northampton, heartily endorsed the proposals of Mr. Battle. Some extremely forcible and interesting forms of appeal, with illustrations, were speedily prepared and circulated, and in the result we are happy to record that the working and other classes of Northampton did in Radium Week subscribe £2,886 at a cost of £226, or, in other words, that the remarkable sum of £2,660 in round figures was subscribed largely by the working classes for the purchase of radium for this hospital. As our technical readers realised when the suggestion was first made, the actual sum raised is none too large to provide sufficient radium to enable Dr. Robson to commence the treatment of suitable cases without avoidable delay.

We congratulate the Mayor of Northampton, and especially the Mayoress, who by organising a Rose Day in connection with Radium Week was instrumental in raising £633, their colleagues and fellow-workers, who represented every class of the inhabitants, upon the remarkable success which has attended their efforts in this matter. Sir Henry Burdett's report upon the General Hospital, Northampton (see The Hospital, February 14, 1914, p. 535), expressed the certainty that if the right steps were taken and sufficient driving force and energy put into the effort, the shortage in the income of this hospital—*i.e.*, £2,000 a year—would be speedily and gladly subscribed by members of the population, to many thousands of whom the General Hospital is indispensable in the day of illness. The success of the Radium Week confirms with telling force the absolute correctness of Sir Henry's forecast in this matter, and we hope his suggestion will be taken to heart and acted upon with promptness by the managers who are responsible for keeping the county hospital adequately supplied with funds.

Mr. Charles Battle is evidently a man of commendable energy and force. He has done a real service not only to the county hospital, but to the working classes, and, indeed, to the whole population of the county of Northampton. Every business to succeed requires skilled direction, organising power, originality, and effort. Mr. Battle has exhibited these qualities and achieved a success which may be the forerunner of many Radium Weeks throughout the United Kingdom, and especially amongst the working classes, who are dependent upon the voluntary hospitals.

Be this as it may, we hope the committee of the General Hospital at Northampton may determine to appoint a financial secretary at a good salary to take up systematically and energetically the work of obtaining all the funds which are required to defray the cost of maintaining the Northampton General Hospital in the highest state of efficiency. This hospital is blessed with a medical staff who have kept the work at the hospital up to date to an extent which does them infinite credit. They are entitled to have placed at their disposal every possible means for expediting the recovery of the patients and securing their freedom from the ravages of disease and the effects of serious illness. The strides taken in the direction of improvements in various directions at this institution in the last ten years redound to the credit of all connected with it, and justify us in expressing the hope that the recent discussion and the present gratifying success of the Radium Week will give the managers sufficient confidence to induce them speedily to adopt the method successfully followed by other great institutions of entrusting the systematic raising of funds to a gentleman possessing similar qualifications and ability to those of Mr. Battle.

In any case we are grateful for the institution of the Radium Week, and its success. It proves that the fault of this financial stress cannot in justice be attributed to any failure on the part of the population to whom the General Hospital ministers. Northampton people of all classes have shown that if a proper appeal is urged upon their attention, and the necessary ability and direction are put behind it, not only will the money be forthcoming, but the personal service of the voluntary workers can be made permanent.

